# From gut to clot: metabolic control of thrombosis by microbiota

**DOI:** 10.1093/lifemeta/loaf025

**Published:** 2025-06-12

**Authors:** Life Metabolism Editorial Team

Hyperactivation of thrombosis is a central pathological process in cardiovascular events such as myocardial infarction and stroke. While antiplatelet therapies remain foundational in clinical prevention, a significant portion of patients continue to experience recurrent thrombotic events, highlighting the urgent need for novel biological targets beyond traditional therapies.

In a recent study published in *Life Metabolism*, Qi and colleagues identified a previously unrecognized link between intermittent fasting (IF), gut microbiota, and platelet hyperactivation [1]. By employing both clinical and preclinical models, the authors demonstrated that IF reduces platelet activation and arterial thrombosis in patients with coronary artery disease (CAD) and in *ApoE*^*–/–*^ mice. Further evidence showed that the anti-thrombotic effect is associated with a marked increase in circulating indole-3-propionic acid (IPA), a gut microbiota-derived metabolite produced by *Clostridium sporogenes* under fasting conditions. Notably, gavage of *C. sporogenes* and IPA treatment could inhibit platelet aggregation and thrombus formation. Mechanistically, IPA was shown to bind directly to the pregnane X receptor (PXR), a nuclear receptor expressed on platelets. This interaction disrupted PXR-mediated pro-thrombotic signaling, thereby attenuating platelet aggregation and thrombus formation.

In a companion study published in *Nature Cardiovascular Research*, the same group uncovered a parallel mechanism involving the gut-derived bile acid and platelet pathway [2]. Bile acids have been known to be metabolized by gut microbiota into bioactive secondary forms such as lithocholic acid (LCA) and deoxycholic acid (DCA). In this study, decreased levels of serum DCA, along with a reduced abundance of *Bacteroides vulgatus*, were found in CAD patients. Furthermore, DCA was found to activate TGR5, a G-protein-coupled receptor expressed on platelets, leading to reduced platelet reactivity and thrombosis. Oral administration of DCA, *B. vulgatus*, or healthy donor stool reduced platelet hyperactivity, thrombosis, and myocardial injury in atherosclerotic mice.

Collectively, these two studies illuminate the gut microbiota as a novel node in thrombosis regulation. By bridging dietary behaviors, microbial metabolism, and platelet activation, these studies pave the way towards more holistic and personalized approaches to cardiovascular prevention.
